# Switching plasmonic nanogaps between classical and quantum regimes with supramolecular interactions

**DOI:** 10.1126/sciadv.abj9752

**Published:** 2022-02-04

**Authors:** Chi Zhang, Dongyao Li, Guangdi Zhang, Xujie Wang, Li Mao, Quan Gan, Tao Ding, Hongxing Xu

**Affiliations:** 1 Key Laboratory of Artificial Micro/Nano Structure of Ministry of Education, School of Physics and Technology, Wuhan University, Wuhan 430072, China.; 2 School of Chemistry and Chemical Engineering, Huazhong University of Science and Technology, Wuhan 430074, China.; 3 School of Microelectronics, Wuhan University, Wuhan 430072, China.

## Abstract

In the realm of extreme nanophotonics, nanogap plasmons support reliable field enhancements up to 1000, which provide unique opportunities to access a single molecule for strong coupling and a single atom for quantum catalysis. The quantum plasmonics are intriguing but difficult to modulate largely because of the lack of proper spacers that can reversibly actuate the sub–1-nm gaps. Here, we demonstrate that supramolecular systems made of oligoamide sequences can reversibly switch the gap plasmons of Au nanoparticles on mirror between classical and quantum tunneling regimes via supramolecular interactions. The results reveal detailed plasmon shift near the quantum tunneling limit, which fits well with both classical- and quantum-corrected models. In the quantum tunneling regime, we demonstrate that plasmonic hot electron tunneling can further blue shift the quantum plasmons because of the increased conductance in the nanogaps, making it a promising prototype of optical tunable quantum plasmonic devices.

## Teaser

A neat switch of metallic nanogaps between insulating and electron tunneling is enabled by molecular machines.

## INTRODUCTION

Plasmonics, because of its wide range of applications in nano-optics (*1*), material science (*2*), biochemical sensing (*3*, *4*), and energy harvesting (*5*), has thrived into a vibrant and interdisciplinary field in decades (*6*). Because many of the superior plasmonic properties are related to their large electric field confinement and small mode volume, engineering the plasmonic nanogaps (hotspots) has been one of the hot topics for nanoplasmonics (*7*). The nanogap plasmon is very sensitive to the gap distance (*d*) with relationship of Δλ ∝ *e*^−*d*^ (*8*), which makes it a plasmonic “ruler” (*9*–*13*) at nanoscale for nanoactuation (*14*), molecular sensing (*15*), chemistry monitoring (*16*, *17*), and thickness determination of ultrathin films (*18*), with resolution that can potentially extend to subpicometer range (*19*). When the gap distance enters the subnanometer scale, the quantum nature of the electrons and the nonlocal screening alter the plasmonic response, which has been theoretically and experimentally exploited (*20*), especially in the regime of “extreme nano-optics” (*21*). However, a grand challenge persists for reversible switching of the nanogaps across the quantum tunneling limit (~0.5 nm), where classical and quantum mechanical models apply (*22*, *23*). The lack of this switching system has rendered limited applications of the quantum plasmonic devices, which is of great interests to the communities (*20*).

A great deal of the reliable fabrication methods that can describe quantum mechanical effects for plasmons in nanogap structures are prompted by the emergence of nanotechnology. Typical top-down approaches such as electron beam irradiation (*24*) and bottom-up approaches based on self-assembly (*25*–*28*) have been the mainstream for decreasing the gap sizes below the quantum tunneling limit. However, the gap size is fixed once the samples are made, which shows poor reversibility. Although “tip-based” gap configurations can be dynamically tuned by spatially controlling the tip-to-tip distance using a modified atomic force microscope (*29*), the critical requirement on sophisticated instrumentation makes this quantum regime not easily accessible in large scale. Electric tuning of the quantum plasmons is more promising for reversible switching with great device compatibility, but it has only been realized in the infrared region so far (*30*, *31*). Although it is suggested theoretically that biased tunneling of the gap conductance can switch the quantum plasmons at optical frequency, the experimental evidence of this concept is yet to be demonstrated (*32*). Optical tuning of the nanogaps below the tunneling limit is more robust and in situ via optical fusion, but this change of gap distance is not reversible (*33*).

**Fig. 1.**
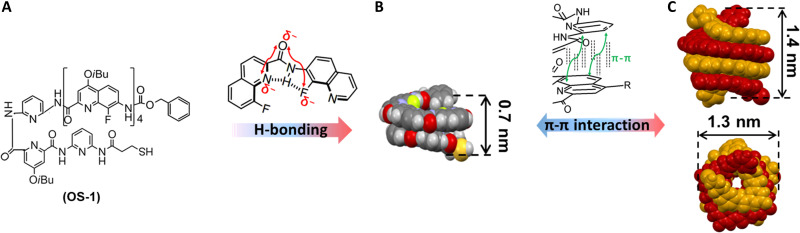
Schematic illustration of the (double) helix formation mechanism. (**A**) Formula of the OS-**1** and (**B**) the folding patterns of the foldamers. H-bonds (dashed lines) and electrostatic repulsions (red arrows) result in a bent conformation, and intramolecular stacking gives rise to a helix. (**C**) Side and top views of the crystal structures of the antiparallel double helix consisting of a pyridine-fluoroquinoline heptamer (*46*).

Supramolecular systems with sizes comparable to the scale of subnanometer provide unique opportunity to facilely access the quantum limit. Several promising supramolecular compounds such as Ln-phthalocyanine complexes (0.3 to 0.4 nm) (*34*) and cucurbiturils (0.9 nm) (*35*) can bring the gap to less than 1 nm, but the plasmonic nanogaps are fixed once formed, as these complexes are rigid and not reconfigurable. Although some of the biological systems such as DNA (*36*–*40*) or DNA origami (*41*, *42*) can be applied to dynamically switch the plasmons, their size is too large (mostly larger than 1 nm) to squeeze into the quantum plasmonic regime. Therefore, an artificially designed system that can change their sizes in angstrom precision is highly desired for the switching of the subnanometer gaps across the quantum tunneling limit. Aromatic oligoamide foldamers are a set of supramolecular system that can potentially be engineered to fit in the size around the nanometer range (*43*). Oligoamide sequence (OS) composed of a pyridine-based trimer and a fluoroquinoline-based tetramer (Fig. 1A) can not only form a single helical structure via intramolecular hydrogen bonding (H-bonding) with a helical length of 0.7 nm (Fig. 1B) but also be subject to dimerizing into an antiparallel double helix with a height of 1.4 nm and a diameter of 1.3 nm (Fig. 1C) (*44*–*46*). The repeating units of the OS can be chemically engineered so that the structure can fit just below the quantum limit (~0.5 nm). Therefore, placing them in the plasmonic nanogaps would benefit enormously for the reversible switching between classical and quantum plasmonic regime, which not only sheds light on quantum plasmonic transitions but also shows important implications for quantum optoelectronic devices.

## RESULTS

**Fig. 2.**
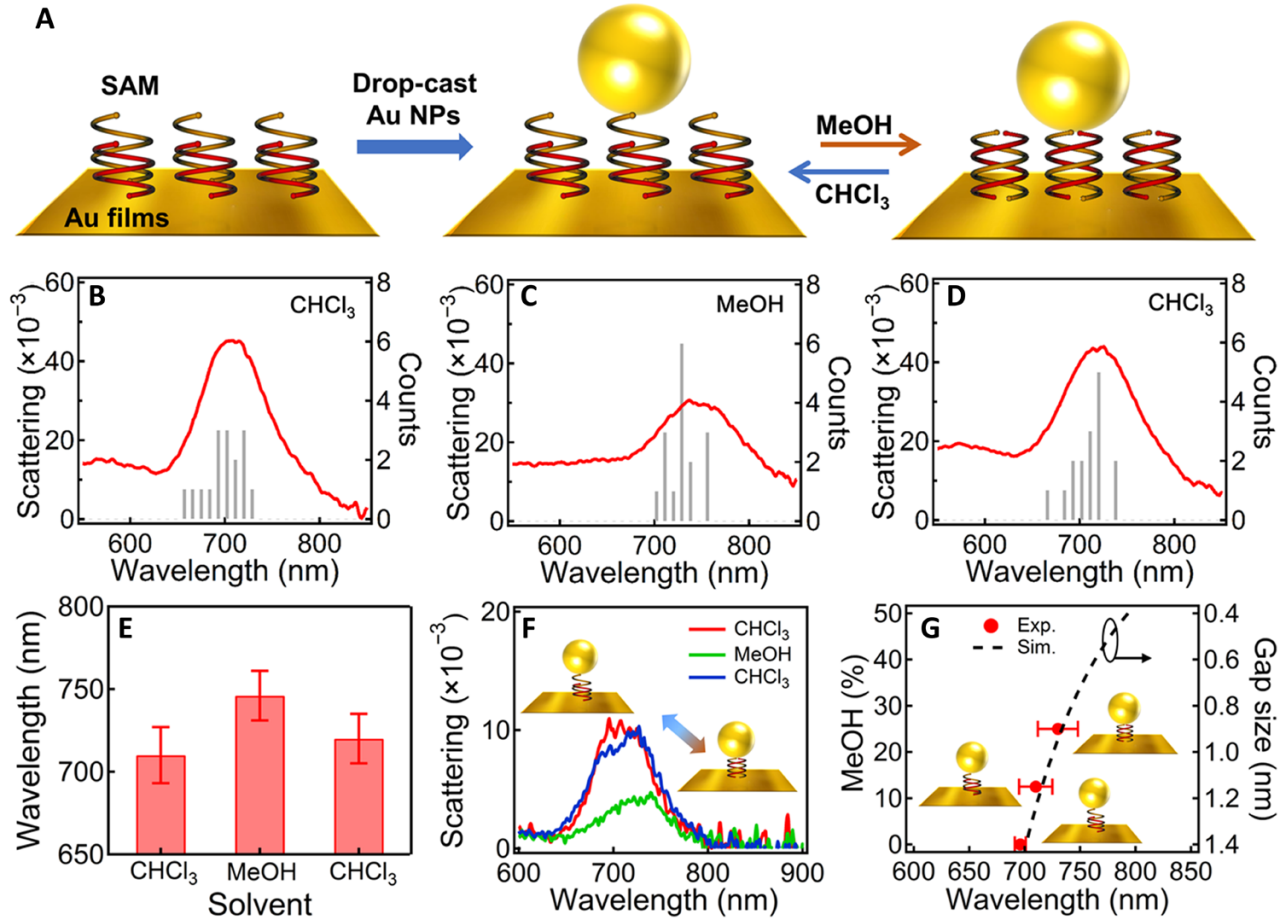
Solvent-induced reversible tuning of Au NPoM plasmons. (**A**) Scheme of the Au NPoM with OS double helices SAM in the nanogap and reversible tuning mechanism. (**B** to **D**) Statistics of the DF scattering spectra of Au NPoMs after incubating in different solvents: (B) CHCl_3_, (C) MeOH, and (D) CHCl_3_. The spectra are collected over 15 randomly selected NPs and averaged. (**E**) Change of the plasmon peak position after incubating in different solvents. (**F**) DF scattering spectra of the same particle after incubating in CHCl_3_, MeOH, and CHCl_3_ again. (**G**) Change of plasmon resonance with MeOH content (red dots) and simulation based on circuit model (black dashed line).

The synthesis of OS-**1** bearing a thiol group at its pyridine terminus is presented in figs. S1 to S7 (*46*). We make a self-assembled monolayer (SAM) of double-helix OS-**1** on Au films followed by drop-casting 80-nm Au nanoparticles (NPs) on top via Au─S bonds (Fig. 2A). Thus, the antiparallel double helices are sandwiched between the Au NPs and the mirror, which can be used to tune the gap sizes via changing their conformations. These Au NPs on mirror (NPoMs) are characterized with dark-field (DF) scattering spectroscopy, which show an averaged peak at ~710 nm (Fig. 2B). This plasmon peak represents the coupled (dipolar) mode of gap plasmons. After being incubated in methanol (MeOH) and vacuum-dried, the plasmon resonances red shift to ~746 nm (Fig. 2C). We attribute this shift of plasmons to the conformational change of the double helix with the change of solvent. Specifically, the arrangement of the double helix may shrink via a spring-like motion to gain more π-π stacking in MeOH (Fig. 2A). This solvent-driven motion is normally observed in solvophobic foldamers (*47*). The original state of the double helix can be recovered if the samples are incubated in CHCl_3_ again, which increases the gap size back to 1.4 nm. Thus, the scattering peaks blue shift back to ~720 nm (Fig. 2D). These scattering spectra are randomly collected over 15 particles and statistically analyzed, so the trend shown in Fig. 2E is rather robust. The variation mainly comes from the slight difference in the sizes and shapes of the Au NPs. We also track the same particle incubated in different solvents, and the scattering spectra are very reversible (Fig. 2F). Again, we can change the polarity by adjusting the solvent ratio of MeOH and CH_2_Cl_2_ to vary the conformations of the double helices so that the gap size of the Au NPoM can be tuned from 1.4 to 0.9 nm, as predicted by SPARTAN (fig. S8) (*48*). Correspondingly, the plasmon resonances red shift from 700 to 730 nm (red points in Fig. 2G and fig. S9), which fits perfectly with the plasmon resonances predicted by the circuit model (dashed line in Fig. 2G) (*49*, *50*).

**Fig. 3.**
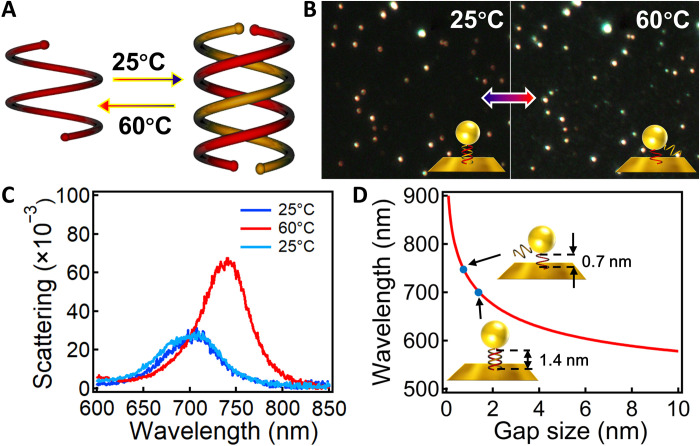
Temperature-induced reversible tuning of Au NPoM plasmons. (**A**) Scheme of the temperature-induced switching of single and double helices. (**B**) DF images of the Au NPoMs after incubation with CHCl_3_ at 25° and 60°C for 1 hour. Insets are the schemes of the corresponding configuration of the OS assemblies in the nanogaps. (**C**) Scattering spectra of the same particle after incubating in chloroform for 1 hour at 25°, 60°, and then back to 25°C. (**D**) Calculated change of plasmon resonance with gap size. Insets indicate the change of OS-**1** in the nanogap from 1.4 to 0.7 nm after heating at 60°C.

Because the equilibrium of the single and double helices of OS is dependent on temperature (*43*), we can further decrease the gap size by disassembling the double helices into single strands when raising the temperature, and this process can be reversed if it cools (Fig. 3A). DF images of the Au NPoMs show prominent change of the scattering colors at different temperatures because of the switch of gap sizes between 0.7 and 1.4 nm (Fig. 3B). The plasmon resonance of the same Au NPoM shows reversible shift between ~705 and ~739 nm, with temperature switching between 25° and 60°C (Fig. 3C). The time-dependent plasmon shift suggests that the dissociation of the double helices completes within 30 s, while it takes much longer time (~3 hours) for them to recombine at room temperature (fig. S10). This thermal-induced plasmon switch can be reproduced for a few cycles (fig. S11), and the deviation in later cycles is mainly due to the slight migration and rotation of the Au NPs on the Au mirror, which makes the plasmon peaks fail to completely recover. Again, by comparing the plasmon shift to the analytical model, the gap size of Au NPoM is suggested to switch between 0.7 and 1.4 nm, which exactly matches the size of single/double-helical structures of OS-**1** (Fig. 3D). This Au NPoM plasmonic ruler provides a facile tool to monitor the assembly and disassembly of the double helices with subangstrom resolution simply by measuring the scattering spectroscopy.

**Fig. 4.**
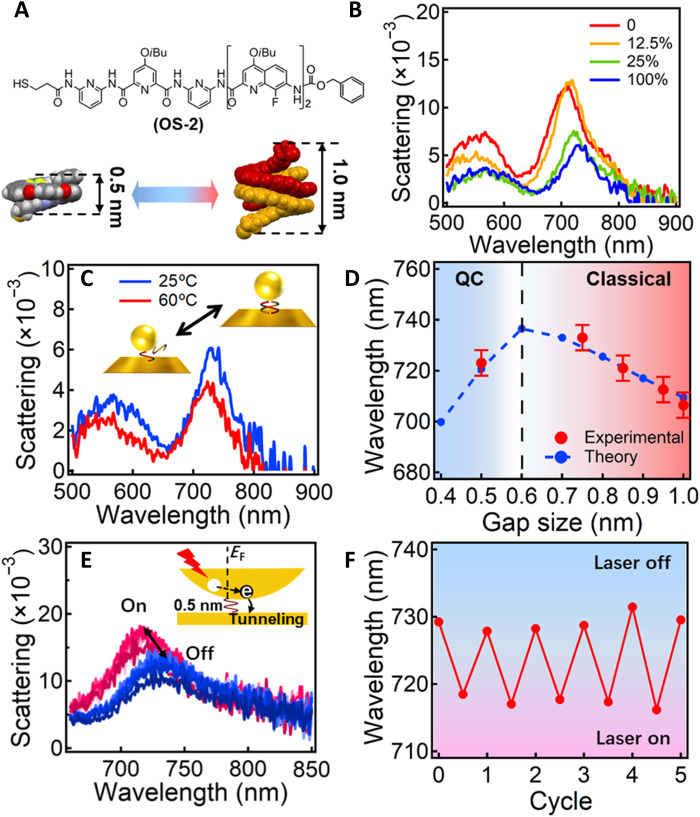
Tuning of the coupled plasmons of Au NPoMs across the classical and quantum regimes. (**A**) Formula of OS-**2** and its configuration of single and double helices. (**B**) Scattering spectra of Au NPoM after incubating in different solvents for 1 hour. The solvents are a mixture of MeOH and CH_2_Cl_2_ with MeOH ratio increasing from 0 to 100%. (**C**) Scattering spectra of the same particle after incubation at 25° and 60°C for 1 hour. (**D**) Change of plasmon resonance with gap size. Red dots are from the experimental, and black lines are from calculation based on quantum-corrected (QC). (**E**) Change of plasmon scattering peak with CW laser (641 nm, 10 μW) on and off for many cycles. Inset scheme illustrates the light-induced hot electron tunneling mechanism, which changes the conductance of the nanogap. (**F**) Shift of plasmon peak with 5 cycles of laser on and off.

To further reduce the gap size below the quantum tunneling limit (*10*, *51*), we choose the shorter OS-**2** with only two fluoroquinoline units as the spacer SAM (Fig. 4A). The OS-**2** forms a single helical strand with a height of 0.5 nm and a duplex with a height of 1.0 nm, which switches right across the quantum tunneling limit. Similarly, with increasing ratio of MeOH and CH_2_Cl_2_, the plasmon resonance red shifts from ~706 to ~733 nm (Fig. 4B), which is, again, because of the contraction of the double helices with increased π-π interactions. This contraction reduces the gap size of Au NPoMs, which results in the red shift of plasmon resonances as classical theory (circuit model) predicts. However, when the gap size is further decreased by fully disassociating the double helices at elevated temperature, the plasmon peak blue shifts to ~723 nm, suggesting a typical feature of quantum plasmonics (Fig. 4C). The peak at ~550 nm is likely the high order mode of Au NPoM, which shows a similar trend of shift as the dipolar mode. The correlation of MeOH content and the double-helix size is simulated with SPARTAN (fig. S8) and used as the gap size of the corresponding plasmon peak. Note that the real gap size may be smaller than the simulated value because of van der Waals compression of the Au; nevertheless, the shift pattern of plasmon peaks basically agrees with the value predicted by the quantum-corrected model (blue dashed line in Fig. 4D), indicating a quantum tunneling limit of around 0.6 nm, which is almost consistent with previous reports (*24*, *26*).

In this quantum tunneling regime, hot electron tunneling driven by laser excitation is also possible, which contributes to the conductance of the nanogaps (inset of Fig. 4E) (*52*, *53*). Thus, we observe a blue shift (~10 nm) of the coupled plasmons upon laser irradiation, which switches back when the laser is turned off (Fig. 4E). Because we use continuous wave (CW) laser with low power (10 μW), the temperature rise in the proximity of the nanogap is only 0.2°C according to the calculations (fig. S12), so the heating effect on the molecular conformation is negligible. This switching is robustly reproduced for many cycles of switching the laser on and off (Fig. 4F), which rules out the possibility of nanogap bridging effect induced by laser irradiation (*54*). In a contrast sample where single-strand OS-**1** (0.7 nm) is used as the gap medium, no such photoswitching of plasmons is observed (fig. S13), as the hot electrons have a very low tunneling rate when the gap size is larger than the tunneling limit (~0.6 nm). The conductivity increase of the nanogaps contributed by the hot electron transfer can be calculated using the formula σ ***=*** *ne*μ, where *n* is the number of hot electrons produced by laser excitation that can be analytically calculated (~4.6 × 10^26^ m^−3^) (*55*), *e* is the unit of electric charge, and μ is the mobility of the hot electrons in OS-**2** molecules, which is estimated to be ~4.2 × 10^−5^ m^2^ V^−1^ s^−1^. Thus, the total conductance increase owing to the hot electrons is ~56G_0_ (G_0_ is the quantum conductance). This increase in conductance predicts a ~11-nm blue shift according to the circuit model, which agrees well with the experiment (Fig. 4, E and F). This small shift of plasmonic scattering represents a spectral characterization of the change of quantum conduction states, which has great implications for quantum optoelectronic devices.

## DISCUSSION

The OS supramolecular systems can be systematically engineered with a series of repeating building blocks that accommodate different gap sizes for different ranges of plasmonic switching. These plasmon shifts fit perfectly with both classical- and quantum-corrected models, which suggest that the quantum limit is approximately 0.6 nm. Reversible photoswitching of the coupled plasmon is also possible in the quantum tunneling regime, where hot electron tunneling via laser excitation increases the gap conductance further. This work provides a facile strategy for reversible tuning of subnanometer gaps with angstrom precision across the quantum limit, which reveals detailed plasmon response at the classical-quantum transition. This supramolecular system based on H-bondings and π-π interactions functions as a nanoactuator for plasmonic nanogaps. The work that goes against the van der Waals potentials between the Au NPs and the mirror is essentially provided by the π-π interactions between the backbone units of the helices. For the nanogap increase from 0.7 to 1.4 nm, the van der Waals potential increases by ~58 kT (fig. S14), while for each double helix of OS-**1**, it contains π-π interaction energy of ~27 kT (see the Supplementary Materials for the calculation). Thus, at least two sets of OS-**1** double helices are formed in the nanogaps to levitate the Au NP 0.7 nm further away from the Au surface. This spring-like actuation of the Au NPoMs works similar to nanojacks, which has great implications for nanomachines. The immediate application of this switchable plasmonic system is to use it as a temperature or solvent sensor by monitoring the shift of plasmon resonances. Besides, the Au NPoM can be a precise plasmonic ruler to monitor the change of OS conformation simply by measuring the scattering spectra of each individual Au NP, which provides a facile and accurate characterization tool for supramolecule chemistry.

## MATERIALS AND METHODS

### Synthesis of OS-1 and OS-2

The compounds OS-**1** and OS-**2** were synthesized through acid chloride-amine condensation reactions followed by the removal of the amino-protecting triphenylmethyl group according to the previous report (*56*) and described with details in fig. S1. The related products are characterized with nuclear magnetic resonance spectroscopy (see figs. S2 to S7).

### Sample preparation

A 3 mm–by–3 mm Au film (70 nm) on Si substrate was immersed in 20 mM OS-**1** (Fig. 1A) or OS-**2** (Fig. 4A)/chloroform solution overnight, followed by rinsing with pure chloroform (99%; Yonghua Chemical Co. Ltd.) and dried quickly. Au NPs (5 μl; 80 nm; BBI Solutions) were drop-casted on the film for 20 min and blow-dried using nitrogen. For solvent treatment, pure chloroform, MeOH (99.8%; Hushi Co. Ltd.) and dichloromethane (99.9%; Aladdin) mixed at the ratio of 1:8 and 1:4, and pure MeOH were used. For temperature-induced disassembly/assembly, the sample was immersed into hot chloroform (60°C) for 30 s and room temperature chloroform for 3 hours, respectively. All the samples were vacuum-dried to remove any residue solvent before taking DF scattering spectra.

### Characterizations

DF scattering spectra of each individual Au NPoM were recorded using a customized DF microscope (BX53, Olympus) through 100× objective (numerical aperture = 0.8) with a 50-μm fiber connected to a fiber spectrometer (QE Pro, Ocean Optics). The Au NPs were selected randomly and tracked for scattering spectroscopy after different solvent and thermal treatments. For light-induced tuning of quantum plasmons, a 641-nm CW laser (Cube, Coherent) was applied to excite the Au NPoMs, and the scattering spectra were collected simultaneously with a 640-nm notch filter before the entrance of spectrometer. The plasmon photoluminescence was subtracted to reveal the change of the white light scattering by the Au NPoMs.

### Simulations

The antenna mode of the Au NPoM is analytically calculated with circuit model on the basis of a simplified formula (*21*)

$$\;\lambda =\lambda_{p}\sqrt{\varepsilon_{\infty }+2\varepsilon_{d}+4\varepsilon_{d}C_{g}/C_{\text{NP}}}$$ (1)

where ε_d_ is the permittivity of the surrounding dielectric medium, and *C*_g_ and *C*_NP_ are the capacitance of the nanogap and the NP, respectively. For spherical NPoM, *C*_g_/*C*_NP =_ ε_g_^0.5^ ln (1 + 0.15*R*/*d*), where *R* and *d* are the radius of the Au NPs and the gap size, respectively. The gap refractive index is set as 1.3 for OS-**1**/OS-**2** with air as the surrounding medium. Calculation in the quantum plasmonic regime is based on the quantum-corrected model of conductivity (*57*)

$$\sigma (l,\omega )=-i\frac{\omega }{4\pi }(\varepsilon (l,\omega )-1)$$ (2)

$$\varepsilon (l,\omega )=\varepsilon_{\infty }-\frac{\omega_{g}^{2}}{\omega (\omega +i\gamma_{g}(l))}$$ (3)

We fit the corrected γ_g_(*l*) according to the time-dependent density functional theory. Considering the presence of OS molecules in the nanogaps, electron hopping mechanism could raise the corrected gap conductance by an order of magnitude larger than that of a vacuum, which is used to calculate the coupled plasmons based on circuit model (fig. S15).

The mobility of hot electrons in the nanogap can be estimated with the formula

$$\mu =T\;\mu_{\text{Au}}$$ (4)

where *T* is the hot electron tunneling probability and μ_Au_ is the electron mobility of bulk Au (see the Supplementary Materials for detailed calculation).

## Supplementary Materials

### This PDF file includes:

Supplementary Text Figs. S1 to S15 References
