# Supplementary material for: Switching plasmonic nanogaps between classical and quantum regimes with supramolecular interactions
Source: Sci Adv. 2022 Feb 4;8(5):eabj9752. doi: 10.1126/sciadv.abj9752 (PMC8816333; doi:10.1126/sciadv.abj9752)
Supplement: Supplementary file 1 — Supplementary Text Figs. S1 to S15 References [file sciadv.abj9752_sm.pdf]

Science Advances
AAAS

Supplementary Materials for

Switching plasmonic nanogaps between classical and quantum regimes with supramolecular interactions

Chi Zhang, Dongyao Li, Guangdi Zhang, Xujie Wang, Li Mao, Quan Gan*, Tao Ding*, Hongxing Xu

*Corresponding author. Email: t.ding@whu.edu.cn (T.D.); ganquan@hust.edu.cn (Q.G.)

Published 4 February 2022, *Sci. Adv.* **8**, eabj9752 (2022)
DOI: 10.1126/sciadv.abj9752

This PDF file includes:

Supplementary Text
Figs. S1 to S15
References

1. Experimental procedures

1.1 General methods

All chemicals and solvents were purchased from commercial suppliers and were used without further purification unless otherwise specified. Dichloromethane (DCM) and diisopropylethylamine (DIEA) was distilled over CaH_2 prior to use. Column chromatography was carried out on Merck GEDURAN Si60 (40-63 μm).

NMR spectra were recorded on Bruker AVANCE 400 (400 MHz) spectrometers. Chemical shifts were calibrated by $CDCl_3$ (7.26 ppm for ^{1}H NMR, 77.16 ppm for ^{13}C NMR) and by DMSO-d_6 (2.50 ppm for ^{1}H NMR, 39.52 ppm for ^{13}C NMR). All chemical shifts (δ) are quoted in ppm and coupling constants (*J*) are expressed in Hertz (Hz). The following abbreviations are used for convenience in reporting the multiplicity for NMR resonances: s = singlet, d = doublet, t = triplet, and m = multiplet. Data processing was performed with Topspin 2.0 software.

High-resolution electrospray ionization mass spectrometry (ESI-MS) was performed on a micro TOF II instrument featuring a Z spray source with electrospray ionization.

1.2 Synthesis of sequences **OS-1** and **OS-2** and NMR characterizations

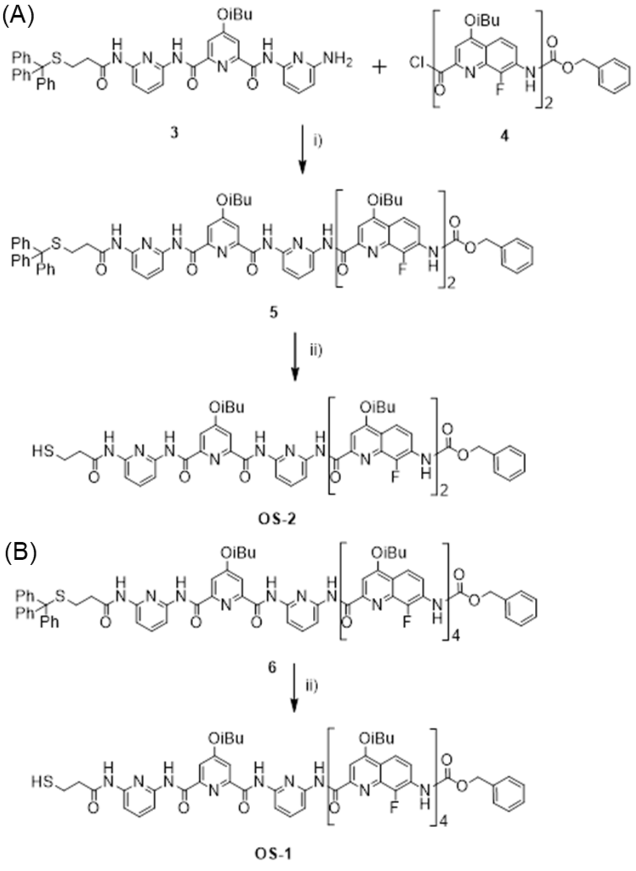

Fig. S1. Synthesis of oligoamide sequences 1 and 2. (A) OS-2: i) DIEA, DCM, room temperature, 12 h. (B) OS-1: ii) Trifluoroacetic acid (TFA), Triisopropylsilane, DCM, room temperature, 3 h.

Compound 5. A solution of the acid chloride **4**[S1] (375 mg, 0.5 mmol) in 4 mL dry DCM was added to a solution of **3**[S1] (350 mg, 0.5 mmol) and DIEA (0.25 mL, 1.5 mmol) in 2 mL dry DCM. The reaction was allowed to proceed overnight. The solvent was removed under reduced pressure. The crude was purified by flash column chromatography (SiO_2) eluting with EtOAc/DCM (1:99 to 50:100) to give the product as a white solid (0.41 g, 58% yield). ^1H NMR (400 MHz, $CDCl_3$): δ 10.67 (s, 1H), 10.45 (s, 1H), 10.32 (s, 1H), 10.04 (s, 1H), 8.65 (s, 1H), 8.35 (s, 1H), 7.95 (d, *J* = 9.2 Hz, 1H), 7.80 (m, 6H), 7.60–7.31 (m, 10H), 7.16 (s, 1H), 7.12–6.89 (m, 15H), 5.24 (s, 2H), 4.09 (d, *J* = 6.0 Hz, 2H), 3.96 (s, 2H), 3.85 (s, 2H), 2.45–2.05 (m, 3H), 2.05 (s, 2H), 1.26 (s, 2H), 1.18 (t, *J* = 6.1 Hz, 12H), 1.10 (d, *J* = 6.7 Hz, 6H). ^{13}C NMR (101 MHz, $CDCl_3$): δ 169.8, 167.8, 163.1, 162.9, 162.4, 161.7, 161.5, 161.1, 153.1, 151.3, 150.63 , 150.2, 149.9, 149.5, 149.3, 149.2, 149.0, 144.4, 140.2, 137.0, 136.0, 129.3, 128.7, 128.3, 127.6, 126.9, 126.3, 120.5, 119.6, 119.0, 118.9, 117.5, 117.4, 112.1, 111.3, 110.5, 109.7, 109.5, 98.1, 97.8, 75.4, 75.2, 67.2, 66.3, 35.2, 28.2, 28.1, 26.5, 19.3, 19.1. ESI-HRMS: m/z calcd for $C_{79}H_{74}F_2N_{11}O_{10}S$ $[M+H]^+$ 1406.5303, found 1406.5309.

OS-2. Under N_2 atmosphere, oligomer **5** (300 mg, 0.16 mmol) were dissolved in DCM:TFA (3:1, 4 mL), and triisopropylsilane (0.22 mL, 1.05 mmol) was added to produce a yellow solution. The reaction mixture was stirred for 2.5 h, then the crude was neutralized with a saturated aqueous solution of $NaHCO_3$, extracted with DCM (3×50 mL). The organic solvent was removed under reduced pressure, and the crude was washed with hexane to give the product as a white solid (230 mg, 91% yield) which was operated without further purification. ^1H NMR (400 MHz, $CDCl_3$): δ 10.62 (s, 1H), 10.44 (s, 1H), 10.41 (s, 1H), 10.19 (s, 1H), 9.33 (s, 1H), 8.68 (t, 1H), 8.37 (t, 1H), 7.98–7.88 (m, 2H), 7.88–7.75 (m, 3H), 7.71 (d, *J* = 12.6 Hz, 2H), 7.62 (t, 1H), 7.51 (s, 1H), 7.38 (m, 7H), 7.18 (s, 1H), 5.20 (s, 2H), 4.11 (d, *J* = 6.5 Hz, 2H), 4.06 (d, 2H), 3.87 (d, *J* = 6.5 Hz, 2H), 2.45–2.26 (m, 4H), 2.18 (m, 3H), 1.25 (s, 1H), 1.20 (d, *J* = 6.7 Hz, 12H), 1.12 (d, *J* = 6.7 Hz, 6H). ^{13}C NMR (101 MHz, $CDCl_3$): δ 170.0, 167.9, 163.1, 162.8, 162.2, 161.6, 161.2, 153.0, 151.0, 150.5, 150.3, 149.7, 149.2, 149.1, 146.9, 146.5, 144.4, 144.0, 140.6, 136.9, 135.8, 128.6, 128.2, 128.0, 126.8, 120.2, 119.5, 119.1, 118.7, 117.6, 117.4, 112.0 , 111.4, 110.3, 110.1, 109.8, 98.0, 97.7, 75.4, 75.3, 67.3, 40.2, 28.2, 28.1, 19.3, 19.1. ESI-HRMS: m/z calcd for $C_{60}H_{60}F_2N_{11}O_{10}S$ $[M+H]^+$ 1164.4208, found 1164.4215.

OS-1. Under N_2 atmosphere, oligomer **6**[S1] (300 mg, 0.16 mmol) were dissolved in DCM: TFA (3:1, 4 mL), and triisopropylsilane (0.16 mL, 0.78 mmol) was added to produce a yellow solution. The reaction mixture was stirred for 2.5 h, then the crude was neutralized with a saturated aqueous solution of $NaHCO_3$, extracted with DCM (3×50 mL). The solvent was removed under reduced pressure, and the crude was washed with hexane to give the product as a white solid (240 mg, 91% yield) which was operated without further purification. ^1H NMR (600 MHz, $CDCl_3$): δ 10.55 (s, 1H), 10.48 (s, 1H), 10.31 (s, 1H), 10.05 (s, 1H), 9.80 (s, 1H), 9.59 (s, 1H), 9.57 (s, 1H), 8.82 (t, 1H), 8.55 (t, 1H), 8.48 (t, 1H), 7.93 (d, *J* = 8.6 Hz, 1H), 7.72 (d, *J* = 7.5 Hz, 1H), 7.66(s, 1H), 7.64 (s, 1H), 7.52 (s, 1H), 7.44–7.32 (m, 4H), 7.27–7.17 (m, 4H), 7.13 (t, 1H), 7.08 (d, 1H), 6.93–6.78 (m, 8H), 4.87 (d, *J* = 12.5 Hz, 1H), 4.25–

4.17 (m, 4H), 4.16–4.09 (m, 4H), 4.06 (t, 1H), 4.00–3.94 (m, 2H), 3.66 (t, 1H), 3.50 (t, 1H), 2.50–2.38 (m, 5H), 2.34 (m, 2H), 2.25 (t, 1H), 1.29–1.21 (m, 18H), 1.09 (d, *J* = 6.5 Hz, 12H). ^{13}C NMR (101 MHz, $CDCl_3$): δ 168.3, 166.2, 161.8, 161.5, 160.8, 160.6, 160.5, 160.3, 160.1, 159.8, 159.5, 151.1, 149.8, 149.6, 149.1, 148.6, 148.5, 148.2, 147.9, 147.5, 147.3, 145.9, 145.6, 145.4, 145.0, 143.4, 143.1, 142.8, 142.5, 138.6, 138.4, 138.1, 135.7, 135.3, 135.3, 133.9, 126.9, 126.6, 126.2, 125.6, 125.3, 118.4, 118.4, 117.9, 117.7, 117.4, 117.3, 117.1, 116.4, 116.1, 115.6, 110.4, 110.2, 109.3, 108.6, 108.1, 96.6, 96.0, 95.7, 74.5, 74.3, 73.9, 73.8, 65.6, 38.8, 27.3, 27.0, 18.3, 18.1. ESI-HRMS: m/z calcd for $C_{88}H_{86}F_4N_{15}O_{14}S$ $[M+H]^+$ 1684.6130, found 1684.6136.

2. Theoretical calculations

2.1 Calculation of hot electron mobility in the nanogaps

The tunneling probability can be calculated using the formula,

$$T = \left[1 + \frac{Sh^2\beta a}{\frac{\frac{\Delta E}{2}+E_F}{W+E_F}\left(1-\frac{\frac{\Delta E}{2}+E_F}{W+E_F}\right)}\right]^{-1} \qquad \text{(S1)}$$

where $\beta = \frac{\sqrt{2m(W-\frac{\Delta E}{2})}}{\hbar}$. Here W is the work function of Au, ΔE is the energy distribution of hot electrons, E_F is the Fermi level of gold, m is the mass of electron, a is the size of the gap. The value of T is estimated to be 0.01.

The electron mobility in bulk Au can be calculated with

$$\mu_{Au} = \frac{\delta}{e \cdot n} \qquad \text{(S2)}$$

$$n = z \cdot \frac{\rho N_A}{M} \qquad \text{(S3)}$$

where δ is the conductivity of Au, z is the number of valence electron of Au, ρ and M are the density and molar mass of Au, respectively.

2.2 Energy calculation of the Au NPoM system with OS-**1**

For the double helix of OS-**1**, it contains ~4 pairs of π-π interaction of pyridine. Since each of π-π interaction in pyridine is ~16.8 kJ/mol (*58*), the total π-π interactions of the OS-**1** double helix is ~67.2 kJ/mol (~27 kT).

For the calculation of Van der Waals interactions between the Au NP and Au films, we adopt simplified model of two Au plate with a separation of d, which can be calculated based on the following formula, which is shown in Fig. S14.

$$U_{vdw} = -\frac{H_{Au}}{6}\left(\frac{2r^2}{d(d+4r)} + \frac{2r^2}{(d+2r)^2} + \ln\frac{d(d+4r)}{(d+2r)^2}\right) \quad \text{(S4)}$$

where H_{Au} is the Hammakker constant of Au (~20), r is the radius of Au NPs (40 nm), d is the separation between the Au NP and Au film.

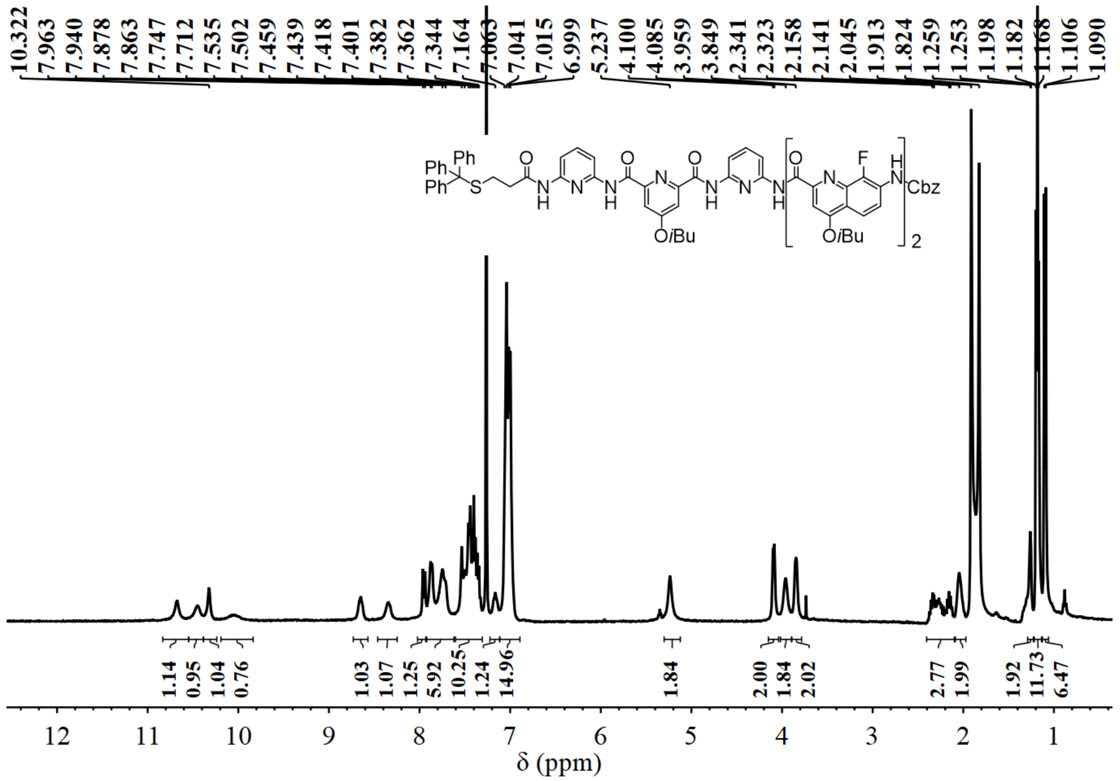

Fig. S2. ^{1}H NMR spectrum (400 MHz) of compound 5 in $CDCl_3$.

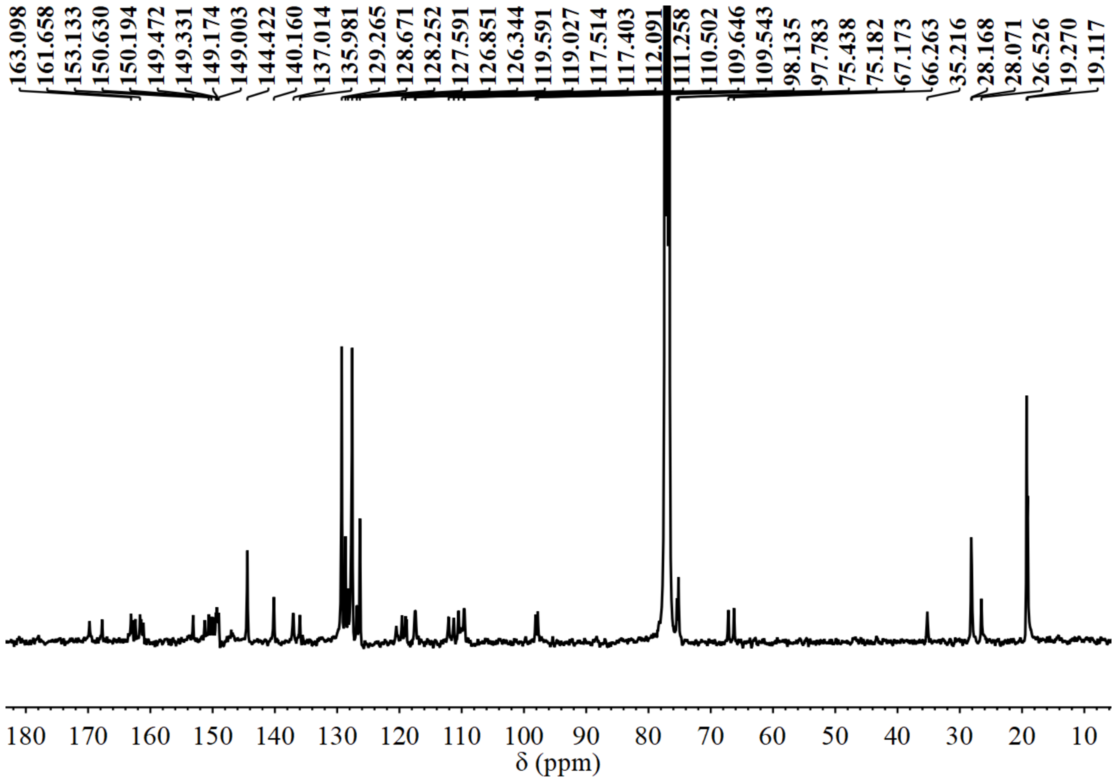

Fig. S3. ^{13}C NMR spectrum (100 MHz) of compound 5 in $CDCl_3$.

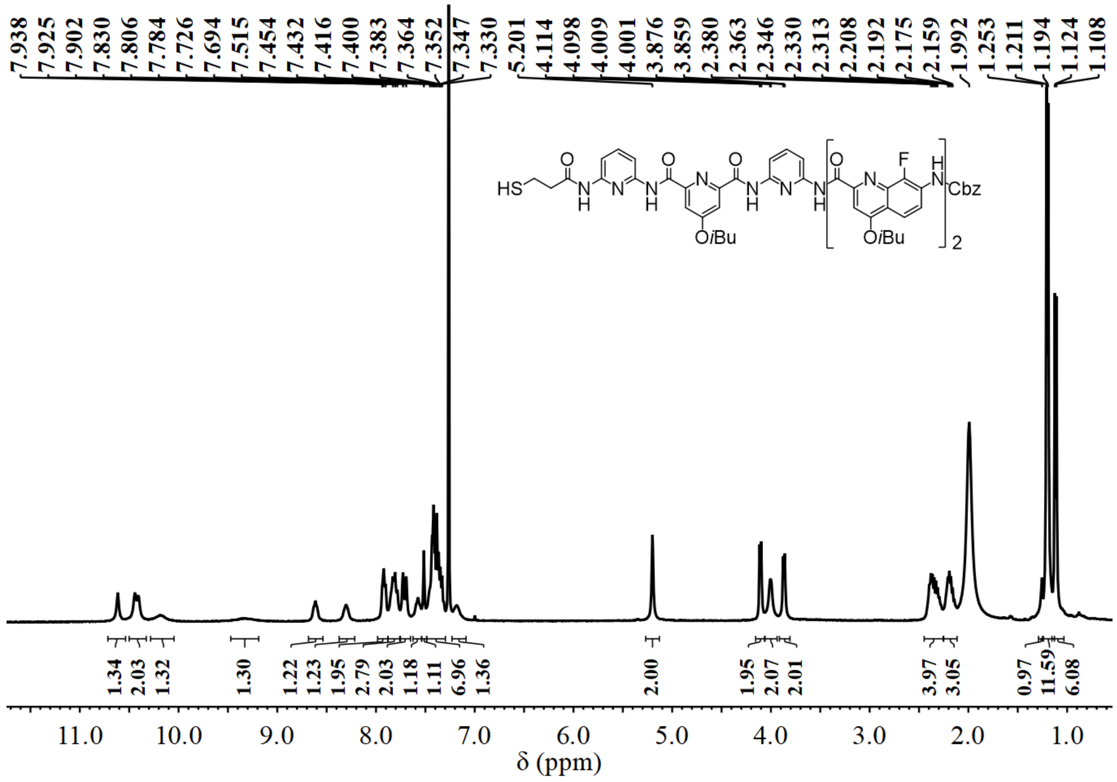

Fig. S4. ^{1}H NMR spectrum (400 MHz) of OS-2 in $CDCl_3$.

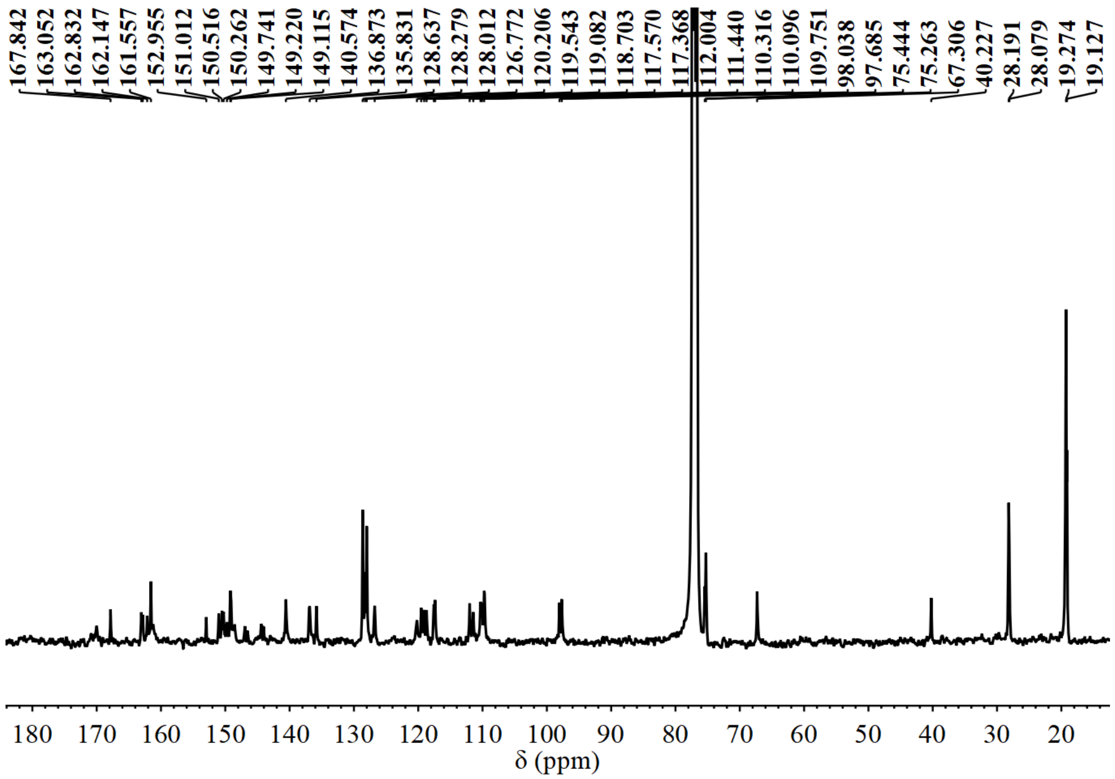

Fig. S5. ^{13}C NMR spectrum (100 MHz) of OS-2 in $CDCl_3$.

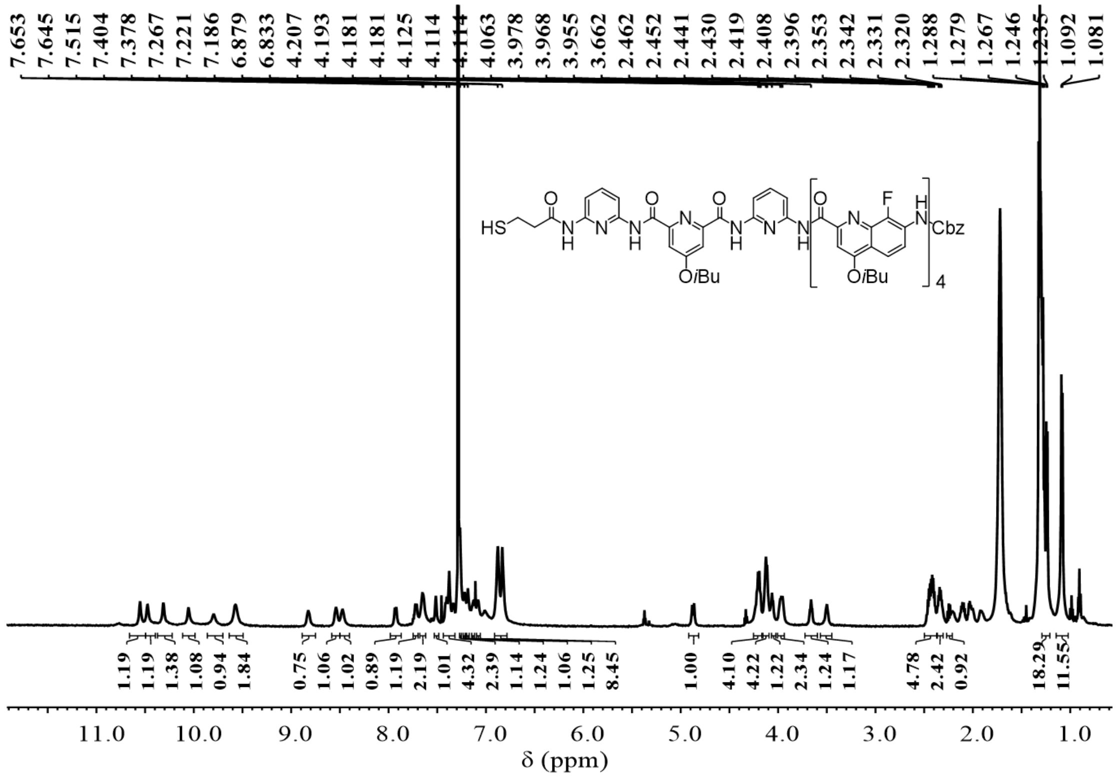

Fig. S6. ^{1}H NMR spectrum (400 MHz) of OS-1 in $CDCl_3$.

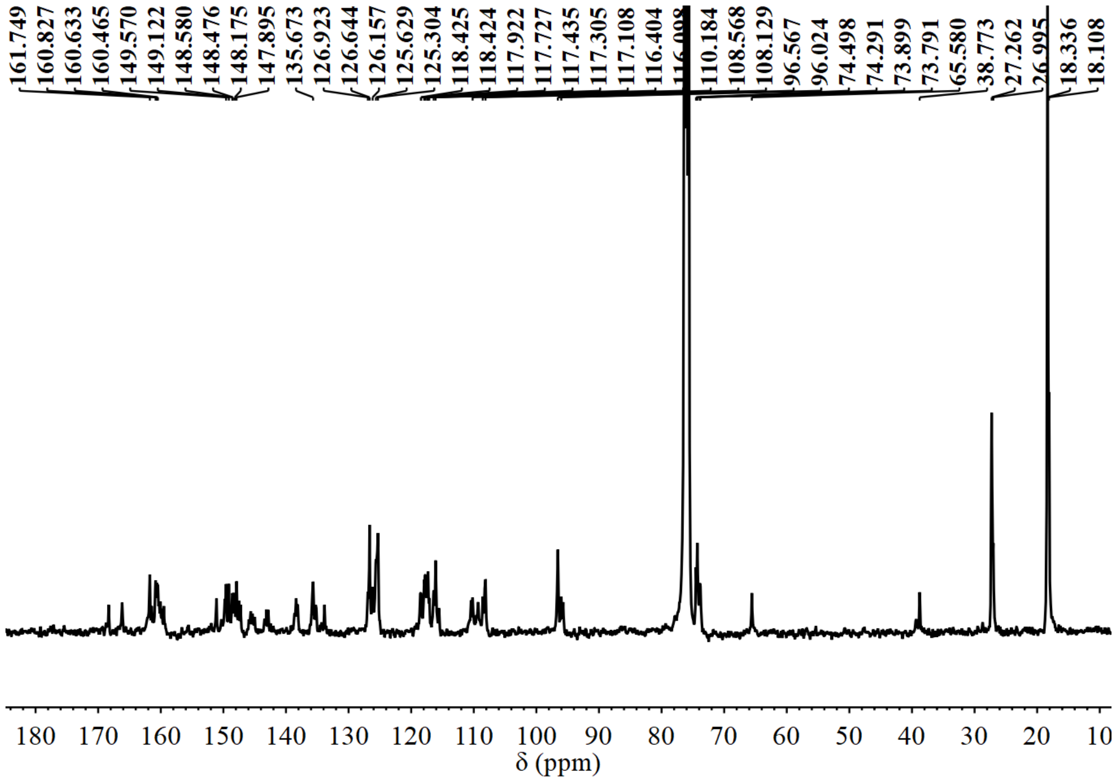

Fig. S7. ^{13}C NMR spectrum (100 MHz) of OS-1 in $CDCl_3$.

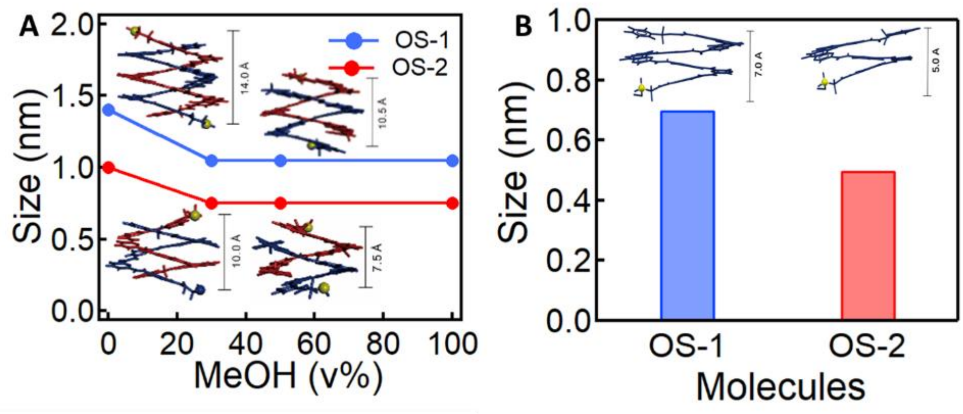

Fig. S8. Molecular simulations with SPARTAN. (A) Change of molecular size with the volume ratio of MeOH, and (B) the sizes of single helical OS-1 and OS-2.

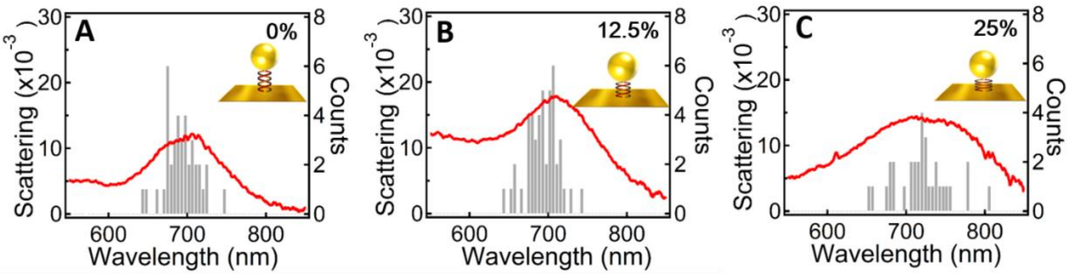

Fig. S9. Solvent-induced tunability of Au NPoM with OS-1 spacer. Scattering spectra of Au NPoM with OS-1 double helix after incubating in different contents of MeOH/CH_2Cl_2. (A) 0%, (B) 12.5%, (C) 25%.

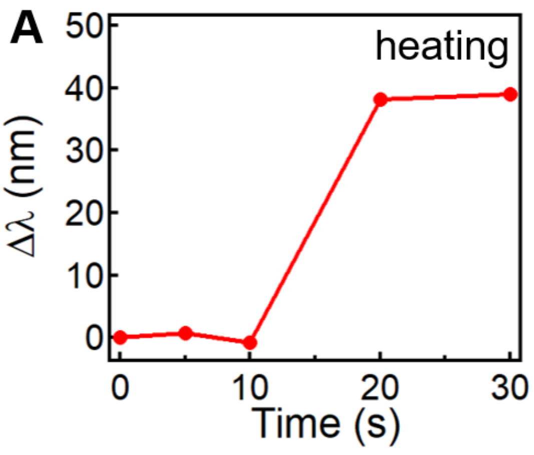

Fig. S10. Assembly and disassembly kinetics of OS-1 double helices. Change of plasmon resonance with the time of heating (A) and cooling (B).

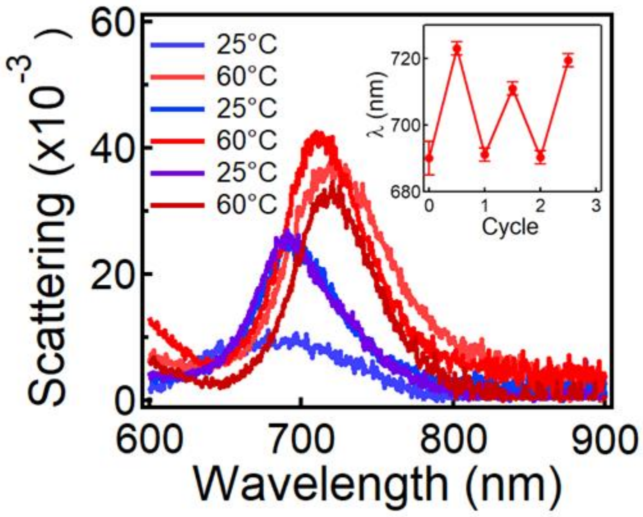

Fig. S11. Reversibility of thermal-induced tuning of Au NPoM with OS-1 as spacer. Scattering spectra of the Au NPoM/OS-1 after cycles of heating and cooling in $CHCl_3$ for 1 h. Inset is the change of plasmon resonance with number of thermal cycles.

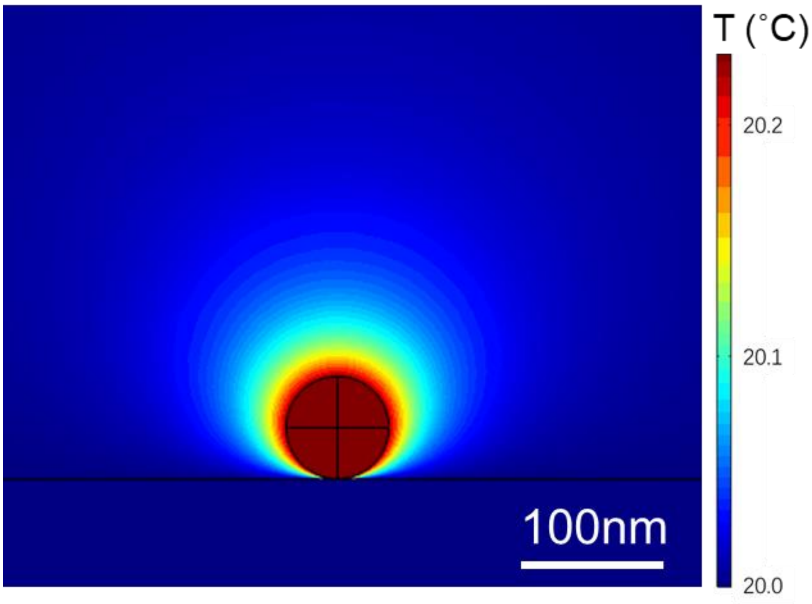

Fig. S12. Temperature distribution around the AuNP under 10 μW laser irradiation.

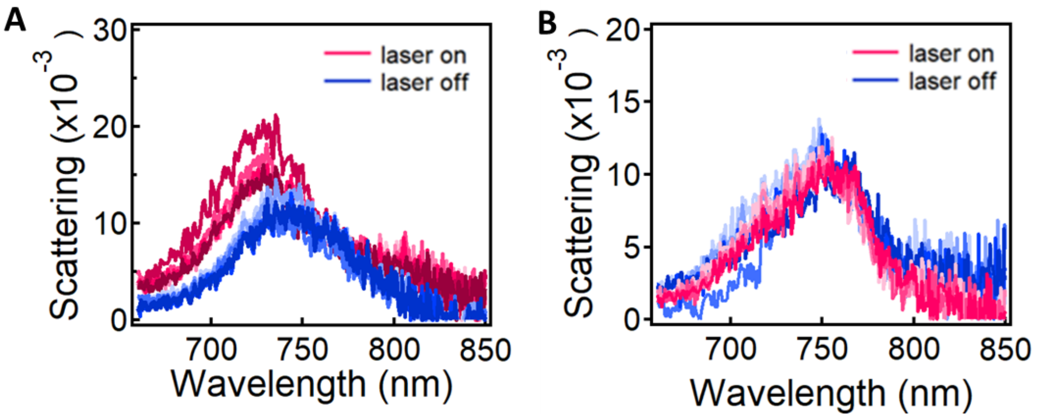

Fig. S13. Laser-induced quantum plasmon switching of Au NPoM. Scattering spectra of (A) Au NPoM/OS-2 (single strand) and (B) Au NPoM/OS-1 (single strand) with laser (641 nm, 10 μW) switched on and off.

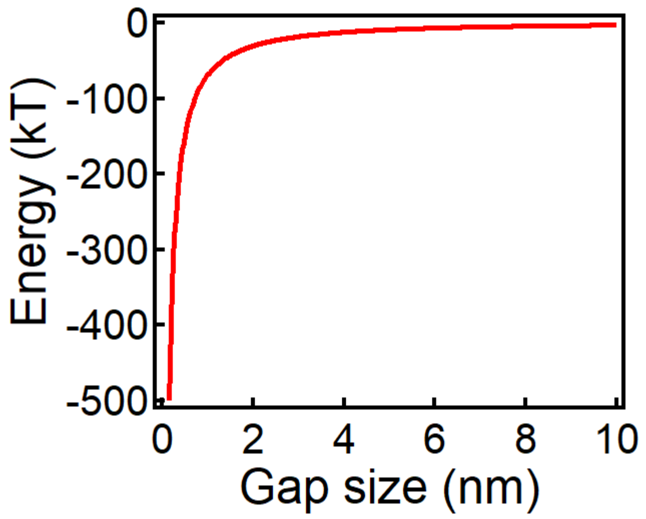

Fig. S14. Theoretical correlation between the Van der Waals potential and gap size supported by double helices of OS-1. When the gap size increases from 0.7 to 1.4 nm, it dissipates energy of ~58 kT, which is equivalent to the π-π interactions of 2 sets of OS-**1** double helices.

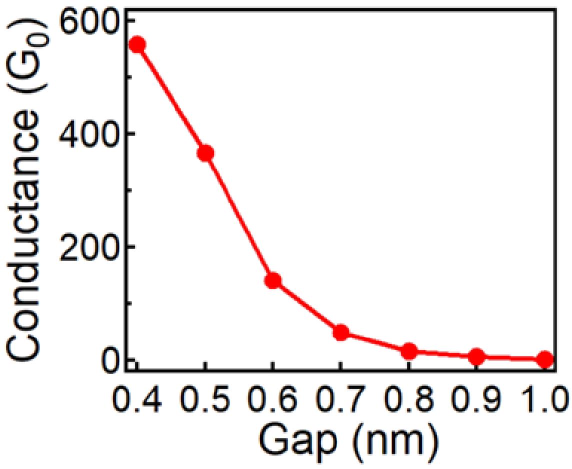

Fig. S15. Calculated change of quantum-corrected gap conductance of Au NPoM/OS-2 with gap size. They are empirically corrected by $170G_0$ for the analytical calculation as suggested by previous literature (*49*).

REFERENCES AND NOTES

1. A. V. Zayats, I. I. Smolyaninov, A. A. Maradudin, Nano-optics of surface plasmon polaritons. *Phys. Rep.* **408**, 131–314 (2005).

2. A. Boltasseva, H. A. Atwater, Low-loss plasmonic metamaterials. *Science* **331**, 290–291 (2011).

3. M. E. Stewart, C. R. Anderton, L. B. Thompson, J. Maria, S. K. Gray, J. A. Rogers, R. G. Nuzzo, Nanostructured plasmonic sensors. *Chem. Rev.* **108**, 494–521 (2008).

4. J. N. Anker, W. P. Hall, O. Lyandres, N. C. Shah, J. Zhao, R. P. Van Duyne, Biosensing with plasmonic nanosensors. *Nat. Mater.* **7**, 442–453 (2008).

5. A. Polman, H. Atwater, Photonic design principles for ultrahigh-efficiency photovoltaics. *Nat. Mater.* **11**, 174–177 (2012).

6. J. A. Schuller, E. S. Barnard, W. Cai, Y. C. Jun, J. S. White, M. L. Brongersma, Plasmonics for extreme light concentration and manipulation. *Nat. Mater.* **9**, 193–204 (2010).

7. P. Gu, W. Zhang, G. Zhang, Plasmonic nanogaps: From fabrications to optical applications. *Adv. Mater. Interfaces* **5**, 1800648 (2018).

8. N. Jiang, X. Zhuo, J. Wang, Active plasmonics: Principles, structures, and applications. *Chem. Rev.* **118**, 3054–3099 (2018).

9. E. W. A. Visser, M. Horáček, P. Zijlstra, Plasmon rulers as a probe for real-time microsecond conformational dynamics of single molecules. *Nano Lett.* **18**, 7927–7934 (2018).

10. R. T. Hill, J. J. Mock, A. Hucknall, S. D. Wolter, N. M. Jokerst, D. R. Smith, A. Chilkoti, Plasmon ruler with angstrom length resolution. *ACS Nano* **6**, 9237–9246 (2012).

11. T. J. Davis, M. Hentschel, N. Liu, H. Giessen, Analytical model of the three-dimensional plasmonic ruler. *ACS Nano* **6**, 1291–1298 (2012).

12. G. R. Bourret, T. Ozel, M. Blaber, C. M. Shade, G. C. Schatz, C. A. Mirkin, Long-range plasmophore rulers. *Nano Lett.* **13**, 2270–2275 (2013).

13. C. Sönnichsen, B. M. Reinhard, J. Liphardt, A. P. Alivisatos, A molecular ruler based on plasmon coupling of single gold and silver nanoparticles. *Nat. Biotechnol.* **23**, 741–745 (2005).

14. D. Schamel, A. G. Mark, J. G. Gibbs, C. Miksch, K. I. Morozov, A. M. Leshansky, P. Fischer, Nanopropellers and their actuation in complex viscoelastic media. *ACS Nano* **8**, 8794–8801 (2014).

15. C. E. Talley, J. B. Jackson, C. Oubre, N. K. Grady, C. W. Hollars, S. M. Lane, T. R. Huser, P. Nordlander, N. J. Halas, Surface-enhanced raman scattering from individual Au nanoparticles and nanoparticle dimer substrates. *Nano Lett.* **5**, 1569–1574 (2005).

16. J. H. Yoon, S. Yoon, Probing interfacial interactions using core–satellite plasmon rulers. *Langmuir* **29**, 14772–14778 (2013).

17. Y.-L. Liu, E. P. Perillo, P. Ang, M. Kim, D. T. Nguyen, K. Blocher, Y.-A. Chen, C. Liu, A. M. Hassan, H. T. Vu, Y.-I. Chen, A. K. Dunn, H.-C. Yeh, Three-dimensional two-color dual-particle tracking microscope for monitoring DNA conformational changes and nanoparticle landings on live cells. *ACS Nano* **14**, 7927–7939 (2020).

18. R. T. Hill, K. M. Kozek, A. Hucknall, D. R. Smith, A. Chilkoti, Nanoparticle–film plasmon ruler interrogated with transmission visible spectroscopy. *ACS Photonics* **1**, 974–984 (2014).

19. W. Chen, S. Zhang, Q. Deng, H. Xu, Probing of sub-picometer vertical differential resolutions using cavity plasmons. *Nat. Commun.* **9**, 801 (2018).

20. W. Zhu, R. Esteban, A. G. Borisov, J. J. Baumberg, P. Nordlander, H. J. Lezec, J. Aizpurua, K. B. Crozier, Quantum mechanical effects in plasmonic structures with subnanometre gaps. *Nat. Commun.* **7**, 11495 (2016).

21. J. J. Baumberg, J. Aizpurua, M. H. Mikkelsen, D. R. Smith, Extreme nanophotonics from ultrathin metallic gaps. *Nat. Mater.* **18**, 668–678 (2019).

22. C. Ciracì, R. T. Hill, J. J. Mock, Y. Urzhumov, A. I. Fernández-Domínguez, S. A. Maier, J. B. Pendry, A. Chilkoti, D. R. Smith, Probing the ultimate limits of plasmonic enhancement. *Science* **337**, 1072–1074 (2012).

23. J. Zuloaga, E. Prodan, P. Nordlander, Quantum description of the plasmon resonances of a nanoparticle dimer. *Nano Lett.* **9**, 887–891 (2009).

24. J. A. Scholl, A. García-Etxarri, A. L. Koh, J. A. Dionne, Observation of quantum tunneling between two plasmonic nanoparticles. *Nano Lett.* **13**, 564–569 (2013).

25. G. Hajisalem, M. S. Nezami, R. Gordon, Probing the quantum tunneling limit of plasmonic enhancement by third harmonic generation. *Nano Lett.* **14**, 6651–6654 (2014).

26. S. F. Tan, L. Wu, J. K. W. Yang, P. Bai, M. Bosman, C. A. Nijhuis, Quantum plasmon resonances controlled by molecular tunnel junctions. *Science* **343**, 1496–1499 (2014).

27. F. Benz, C. Tserkezis, L. O. Herrmann, B. de Nijs, A. Sanders, D. O. Sigle, L. Pukenas, S. D. Evans, J. Aizpurua, J. J. Baumberg, Nanooptics of molecular-shunted plasmonic nanojunctions. *Nano Lett.* **15**, 669–674 (2015).

28. H. Cha, J. H. Yoon, S. Yoon, Probing quantum plasmon coupling using gold nanoparticle dimers with tunable interparticle distances down to the subnanometer range. *ACS Nano* **8**, 8554–8563 (2014).

29. K. J. Savage, M. M. Hawkeye, R. Esteban, A. G. Borisov, J. Aizpurua, J. J. Baumberg, Revealing the quantum regime in tunnelling plasmonics. *Nature* **491**, 574–577 (2012).

30. X. Liu, J.-H. Kang, H. Yuan, J. Park, S. J. Kim, Y. Cui, H. Y. Hwang, M. L. Brongersma, Electrical tuning of a quantum plasmonic resonance. *Nat. Nanotechnol.* **12**, 866–870 (2017).

31. A. N. Grigorenko, M. Polini, K. S. Novoselov, Graphene plasmonics. *Nat. Photonics* **6**, 749–758 (2012).

32. D. C. Marinica, M. Zapata, P. Nordlander, A. K. Kazansky, P. M. Echenique, J. Aizpurua, A. G. Borisov, Active quantum plasmonics. *Sci. Adv.* **1**, e1501095 (2015).

33. H. Jung, H. Cha, D. Lee, S. Yoon, Bridging the nanogap with light: Continuous tuning of plasmon coupling between gold nanoparticles. *ACS Nano* **9**, 12292–12300 (2015).

34. C. Readman, B. de Nijs, I. Szabó, A. Demetriadou, R. Greenhalgh, C. Durkan, E. Rosta, O. A. Scherman, J. J. Baumberg, Anomalously large spectral shifts near the quantum tunnelling limit in plasmonic rulers with subatomic resolution. *Nano Lett.* **19**, 2051–2058 (2019).

35. E. Masson, X. Ling, R. Joseph, L. Kyeremeh-Mensah, X. Lu, Cucurbituril chemistry: A tale of supramolecular success. *RSC Adv.* **2**, 1213–1247 (2012).

36. M. P. Busson, B. Rolly, B. Stout, N. Bonod, E. Larquet, A. Polman, S. Bidault, Optical and topological characterization of gold nanoparticle dimers linked by a single DNA double strand. *Nano Lett.* **11**, 5060–5065 (2011).

37. S. Samai, T. L. Y. Choi, K. N. Guye, Y. Yan, D. S. Ginger, Plasmonic nanoparticle dimers with reversibly photoswitchable interparticle distances linked by DNA. *J. Phys. Chem. C* **122**, 13363–13370 (2018).

38. N. Liu, T. Liedl, DNA-assembled advanced plasmonic architectures. *Chem. Rev.* **118**, 3032–3053 (2018).

39. L. Lermusiaux, S. Bidault, Temperature-dependent plasmonic responses from gold nanoparticle dimers linked by double-stranded DNA. *Langmuir* **34**, 14946–14953 (2018).

40. K. Lee, V. P. Drachev, J. Irudayaraj, DNA−gold nanoparticle reversible networks grown on cell surface marker sites: Application in diagnostics. *ACS Nano* **5**, 2109–2117 (2011).

41. B. Shen, M. A. Kostiainen, V. Linko, DNA origami nanophotonics and plasmonics at interfaces. *Langmuir* **34**, 14911–14920 (2018).

42. K. Martens, F. Binkowski, L. Nguyen, L. Hu, A. O. Govorov, S. Burger, T. Liedl, Long- and short-ranged chiral interactions in DNA-assembled plasmonic chains. *Nat. Commun.* **12**, 2025 (2021).

43. D.-W. Zhang, X. Zhao, J.-L. Hou, Z.-T. Li, Aromatic amide foldamers: Structures, properties, and functions. *Chem. Rev.* **112**, 5271–5316 (2012).

44. Q. Gan, Y. Ferrand, C. Bao, B. Kauffmann, A. Grélard, H. Jiang, I. Huc, Helix-rod host-guest complexes with shuttling rates much faster than disassembly. *Science* **331**, 1172–1175 (2011).

45. Q. Gan, C. Bao, B. Kauffmann, A. Grélard, J. Xiang, S. Liu, I. Huc, H. Jiang, Quadruple and double helices of 8-fluoroquinoline oligoamides. *Angew. Chem. Int. Ed.* **47**, 1715–1718 (2008).

46. D. Li, C. Ma, J. Xiang, K. Zhang, L. Yang, Q. Gan, A disulfide switch providing absolute handedness control in double helices via conversion from the antiparallel to parallel helical pattern. *Chem. Eur. J.* **27**, 11663–11669 (2021).

47. T. Qi, V. Maurizot, H. Noguchi, T. Charoenraks, B. Kauffmann, M. Takafuji, H. Ihara, I. Huc, Solvent dependence of helix stability in aromatic oligoamide foldamers. *Chem. Commun.* **48**, 6337–6339 (2012).

48. W. J. Hehre, *A Guide to Molecular Mechanics and Quantum Chemical Calculations* (Wavefunction Inc., 2014).

49. F. Benz, B. de Nijs, C. Tserkezis, R. Chikkaraddy, D. O. Sigle, L. Pukenas, S. D. Evans, J. Aizpurua, J. J. Baumberg, Generalized circuit model for coupled plasmonic systems. *Opt. Express* **23**, 33255–33269 (2015).

50. C. Tserkezis, R. Esteban, D. O. Sigle, J. Mertens, L. O. Herrmann, J. J. Baumberg, J. Aizpurua, Hybridization of plasmonic antenna and cavity modes: Extreme optics of nanoparticle-on-mirror nanogaps. *Phys. Rev. A* **92**, 053811 (2015).

51. W. Zhu, K. B. Crozier, Quantum mechanical limit to plasmonic enhancement as observed by surface-enhanced Raman scattering. *Nat. Commun.* **5**, 5228 (2014).

52. N. Ittah, Y. Selzer, Electrical detection of surface plasmon polaritons by $1G_0$ gold quantum point contacts. *Nano Lett.* **11**, 529–534 (2011).

53. D. R. Ward, F. Hüser, F. Pauly, J. C. Cuevas, D. Natelson, Optical rectification and field enhancement in a plasmonic nanogap. *Nat. Nanotechnol.* **5**, 732–736 (2010).

54. J. Mertens, A. Demetriadou, R. W. Bowman, F. Benz, M. E. Kleemann, C. Tserkezis, Y. Shi, H. Y. Yang, O. Hess, J. Aizpurua, J. J. Baumberg, Tracking optical welding through groove modes in plasmonic nanocavities. *Nano Lett.* **16**, 5605–5611 (2016).

55. L. V. Besteiro, X.-T. Kong, Z. Wang, G. Hartland, A. O. Govorov, Understanding hot-electron generation and plasmon relaxation in metal nanocrystals: Quantum and classical mechanisms. *ACS Photonics* **4**, 2759–2781 (2017).

56. P. Mateus, A. Jacquet, A. Méndez-Ardoy, A. Boulloy, B. Kauffmann, G. Pecastaings, T. Buffeteau, Y. Ferrand, D. M. Bassani, I. Huc, Sensing a binding event through charge transport variations using an aromatic oligoamide capsule. *Chem. Sci.* **12**, 3743–3750 (2021).

57. R. Esteban, A. G. Borisov, P. Nordlander, J. Aizpurua, Bridging quantum and classical plasmonics with a quantum-corrected model. *Nat. Commun.* **3**, 825 (2012).

58. B. K. Mishra, N. Sathyamurthy, π−π interaction in pyridine. *J. Phys. Chem. A* **109**, 6–8 (2005).
